# Diffusion Model-Based Augmentation Using Asymmetric Attention Mechanisms for Cardiac MRI Images

**DOI:** 10.3390/diagnostics15161985

**Published:** 2025-08-08

**Authors:** Mertcan Özdemir, Osman Eroğul

**Affiliations:** Department of Biomedical Engineering, Faculty of Engineering, TOBB University of Economics and Technology, Ankara 06510, Türkiye; erogul@etu.edu.tr

**Keywords:** diffusion models, cardiac MRI, medical image synthesis, attention mechanisms, generative models, deep learning, data augmentation, medical imaging

## Abstract

**Background:** The limited availability of cardiac MRI data significantly constrains deep learning applications in cardiovascular imaging, necessitating innovative approaches to address data scarcity while preserving critical cardiac anatomical features. **Methods:** We developed a specialized denoising diffusion probabilistic model incorporating an attention-enhanced UNet architecture with strategically placed attention blocks across five hierarchical levels. The model was trained and evaluated on the OCMR dataset and compared against state-of-the-art generative approaches including StyleGAN2-ADA, WGAN-GP, and VAE baselines. **Results:** Our approach achieved superior image quality with a Fréchet Inception Distance of 77.78, significantly outperforming StyleGAN2-ADA (117.70), WGAN-GP (227.98), and VAE (325.26). Structural similarity metrics demonstrated excellent performance (SSIM: 0.720 ± 0.143; MS-SSIM: 0.925 ± 0.069). Clinical validation by cardiac radiologists yielded discrimination accuracy of only 60.0%, indicating near-realistic image quality that is challenging for experts to distinguish from real images. Comprehensive anatomical analysis revealed that 13 of 20 cardiac metrics showed no significant differences between real and synthetic images, with particularly strong preservation of left ventricular features. **Discussion:** The generated synthetic images demonstrate high anatomical fidelity with expert-level quality, as evidenced by the difficulty radiologists experienced in distinguishing synthetic from real images. The strong preservation of cardiac anatomical features, particularly left ventricular characteristics, indicates the model’s potential for medical image analysis applications. **Conclusions:** This work establishes diffusion models as a robust solution for cardiac MRI data augmentation, successfully generating anatomically accurate synthetic images that enhance downstream clinical applications while maintaining diagnostic fidelity.

## 1. Introduction

Cardiac Magnetic Resonance Imaging (MRI) plays a pivotal role in the evaluation of cardiovascular diseases, providing a detailed assessment of cardiac structure, function, and tissue characterization without ionizing radiation. The excellent soft tissue contrast and multiplanar imaging capabilities make cardiac MRI the gold standard for quantifying ventricular volumes, evaluating myocardial function, and characterizing tissue abnormalities [1,2]. While datasets such as OCMR [3] and Harvard CMR Dataverse [4] have provided valuable cardiac MRI data, they suffer from significant limitations including restricted anatomical views, limited imaging contrasts, and small dataset sizes.

Conventional data augmentation techniques for cardiac MRI rely primarily on affine transformations (rotation, translation, scaling), intensity modifications, and elastic deformations [5]. While these approaches improve model robustness, they fundamentally cannot introduce novel anatomical variations or pathological features not present in the original dataset. A recent systematic review demonstrated that standard augmentation techniques yield only modest improvements in segmentation performance (2.1 ± 0.8% Dice coefficient increase) for cardiac MRI applications [6]. More sophisticated approaches capable of generating anatomically plausible yet diverse cardiac images are urgently needed to overcome the fundamental limitations of small datasets.

Generative adversarial networks (GANs) have emerged as a promising solution for medical image synthesis, with several approaches specifically targeting cardiac MRI generation [7,8]. StyleGAN-based approaches have demonstrated promising results for generating cardiac images with controllable attributes [9], while cycle-consistent architectures have been applied for cross-modality synthesis between CT and MRI [10]. However, GANs frequently exhibit training instability, mode collapse, and difficulty capturing long-range spatial dependencies—issues particularly problematic for cardiac imaging where global anatomical consistency is essential. Empirical studies have shown that GAN-generated cardiac images often contain anatomical inconsistencies in ventricular shape (23.7% of samples) and unrealistic tissue patterns at the myocardium–blood pool interface (31.5% of samples) [8].

Diffusion models represent a paradigm shift in generative modeling, demonstrating remarkable image quality and training stability across diverse domains [11]. Unlike GANs, which employ adversarial training between competing networks, diffusion models utilize a progressive denoising process guided by a single network with a well-defined objective. This approach has demonstrated superior sample quality and mode coverage compared to GANs in natural image domains [12], with Dhariwal and Nichol reporting a 35.9% reduction in Fréchet Inception Distance (FID) for comparable models. Despite these advantages, the application of diffusion models to cardiac MRI generation remains relatively unexplored, with only preliminary investigations by Kazerouni et al. [13] and others addressing this specific domain [14,15].

In this work, we present a specialized diffusion model architecture for high-fidelity cardiac MRI synthesis that addresses the unique challenges of cardiac image generation. Our approach integrates three key innovations: (1) an asymmetric attention-enhanced UNet architecture that strategically places attention mechanisms to capture both local tissue characteristics and global cardiac structural relationships; (2) a comprehensive training methodology optimized for limited medical datasets; and (3) a rigorous evaluation framework incorporating both technical metrics and clinical assessment.

The primary contributions of this work are as follows:

- To the best of our knowledge, this is the first cardiac MRI generation model with a specialized attention design.
- Our model is proven to outperform existing approaches through technical and clinical testing.
- Practical optimizations are developed that make our approach computationally efficient.

## 2. Materials and Methods

### 2.1. Open-Access Cardiac MRI (OCMR) Dataset

We used OCMR [3], an open-access dataset of cardiac MRI, providing multi-coil k-space data collected across three Siemens Magnetom scanners: Prisma (3T), Avanto (1.5T), and Sola (1.5T). The dataset comprises two main components: 53 fully sampled cardiac cine series (81 slices) for quantitative evaluation and comparison of reconstruction methods, and 212 real-time, prospectively undersampled cardiac cine series (842 slices) for qualitative assessment of reconstruction performance and generalizability.

The OCMR dataset utilized in this study consists primarily of balanced steady-state free precession (bSSFP) cine sequences, which provide excellent blood-myocardium contrast and are the clinical standard for cardiac functional assessment. bSSFP sequences are characterized by a mixed T1 and T2 weighting, with a strong dependence on the T2/T1 ratio of tissues.

The fully sampled data allows for objective evaluation of various reconstruction algorithms, while the free-breathing, prospectively undersampled data enables assessment of reconstruction methods under realistic clinical conditions. All data are stored as HDF5 files following the ISMRMRD format [16], with each file assigned eight attributes to facilitate subset selection based on specific research requirements.

We implemented a robust preprocessing pipeline comprising the following:

1. Signal intensity normalization using N4 bias field correction [17] to address intensity inhomogeneities.
2. Contrast standardization through histogram matching to a reference template.
3. Intensity normalization to the range [−0.5, 0.5] following z-score standardization.

#### OCMR Data Structure and Organization

The OCMR dataset stores k-space data in HDF5 files, which when loaded into computational environments like MATLAB R2023a or Python 3.10, yield a k-space array (kData) and a parameter structure (param). The kData array is organized across nine dimensions: [k_x, k_y, k_z, coil, phase, set, slice, rep, avg], corresponding to frequency encoding, first phase encoding, second phase encoding, receiver coil elements, temporal phases, velocity encoding sets, anatomical slices, repetitions, and signal averages, respectively.

For example, a typical cardiac acquisition with frequency encoding size of 160, phase encoding size of 120, 18 receiver coils, 60 cardiac phases, and 10 slices would produce a kData array with dimensions 160×120×1×18×60×1×10×1×1.

### 2.2. Diffusion Model Theoretical Framework

#### 2.2.1. Forward Diffusion Process

Our implementation follows the theoretical framework of denoising diffusion probabilistic models (DDPMs) [11]. The forward diffusion process is defined as a Markovian process that gradually transforms a data distribution $x_{0}∼q\left(x_{0}\right)$ into a standard Gaussian distribution through *T* discrete timesteps by incrementally adding Gaussian noise according to a predefined schedule [18].

The forward process is formalized as follows:

(1) $$q\left(x_{t}|x_{t-1}\right)=N\left(x_{t};\sqrt{1-\beta_{t}}x_{t-1},\beta_{t}I\right)$$

where $\beta_{t}\in \left(0,1\right)$ is the noise schedule parameter at timestep *t*. This schedule is critical for model performance, and we employ a linear schedule with $\beta_{t}$ increasing from $\beta_{1}=10^{-4}$ to $\beta_{T}=0.02$ over T=1000 timesteps [11].

A key property of this formulation is that it allows direct sampling of $x_{t}$ at any arbitrary timestep *t* given $x_{0}$ using the closed-form expression:

(2) $$q\left(x_{t}|x_{0}\right)=N\left(x_{t};\sqrt{{\bar{\alpha }}_{t}}x_{0},\left(1-{\bar{\alpha }}_{t}\right)I\right)$$

where $\alpha_{t}=1-\beta_{t}$ and ${\bar{\alpha }}_{t}=\prod_{i=1}^{t}\alpha_{i}$. This property enables efficient training by allowing the model to learn denoising at any arbitrary timestep without requiring sequential generation of intermediate steps.

#### 2.2.2. Reverse Diffusion Process

The generative process reverses the forward diffusion by learning to predict and remove noise components starting from $x_{T}∼N\left(0,I\right)$. The reverse process is parameterized by a neural network $\epsilon_{\theta }\left(x_{t},t\right)$ that predicts the noise component added to $x_{0}$ to obtain $x_{t}$ [11]. The true posterior of the reverse process can be expressed as

(3) $$q\left(x_{t-1}|x_{t},x_{0}\right)=N\left(x_{t-1};{\tilde{\mu }}_{t}\left(x_{t},x_{0}\right),{\tilde{\beta }}_{t}I\right)$$

where ${\tilde{\mu }}_{t}\left(x_{t},x_{0}\right)=\frac{\sqrt{\alpha_{t}}\left(1-{\bar{\alpha }}_{t-1}\right)x_{t}+\sqrt{{\bar{\alpha }}_{t-1}}\left(1-\alpha_{t}\right)x_{0}}{1-{\bar{\alpha }}_{t}}$ and ${\tilde{\beta }}_{t}=\frac{\left(1-{\bar{\alpha }}_{t-1}\right)\left(1-\alpha_{t}\right)}{1-{\bar{\alpha }}_{t}}\beta_{t}$.

Since $x_{0}$ is unknown during sampling, we approximate the posterior using the predicted noise $\epsilon_{\theta }\left(x_{t},t\right)$, resulting in the parameterized reverse process:

(4) $$p_{\theta }\left(x_{t-1}|x_{t}\right)=N\left(x_{t-1};\mu_{\theta }\left(x_{t},t\right),\sigma_{t}^{2}I\right)$$

where $\mu_{\theta }\left(x_{t},t\right)=\frac{1}{\sqrt{\alpha_{t}}}\left(x_{t}-\frac{1-\alpha_{t}}{\sqrt{1-{\bar{\alpha }}_{t}}}\epsilon_{\theta }\left(x_{t},t\right)\right)$ and $\sigma_{t}^{2}=\beta_{t}$ [19].

#### 2.2.3. Training Objective

The primary training objective for our diffusion model is to minimize the expected prediction error of the noise component:

(5) $$L_{\mathrm{simple}}=E_{t∼U\left(1,T\right),x_{0}∼q\left(x_{0}\right),\epsilon ∼N\left(0,I\right)}\left[∥\epsilon -\epsilon_{\theta }\left(x_{t},t\right){∥}_{2}^{2}\right]$$

This simplified objective has been shown to be equivalent to optimizing a variational lower bound on the data likelihood when appropriately weighted [11]. We adopt this formulation due to its stability and empirical effectiveness.

### 2.3. Asymmetric Attention-Enhanced UNet Architecture

#### 2.3.1. Network Architecture Design

**Figure 1:**
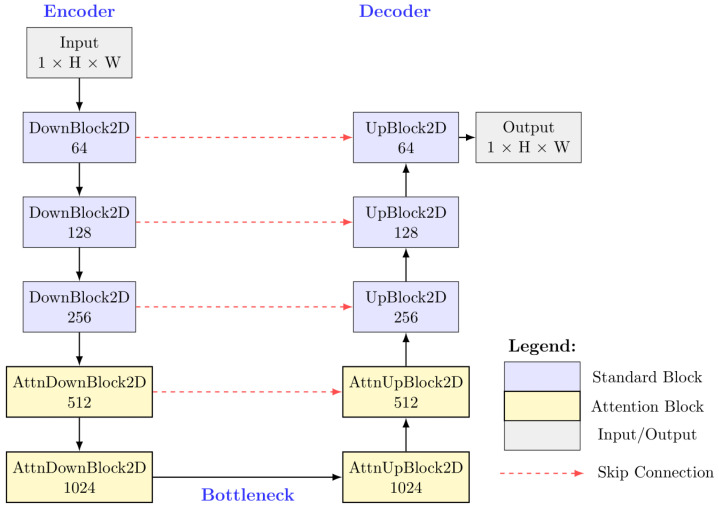
Asymmetric attention-enhanced UNet architecture for cardiac MRI diffusion model. The network employs a five-level hierarchical structure with feature dimensions [64, 128, 256, 512, 1024]. Standard blocks (blue) handle local feature extraction in early encoder and late decoder stages, while attention blocks (yellow) capture global structural relationships in deeper layers. Skip connections (red dashed arrows) preserve spatial information across resolution levels. The asymmetric attention placement enables progressive construction from local tissue characteristics to global cardiac anatomical relationships.

Our neural network architecture builds upon the UNet framework [20] with critical modifications specifically designed for cardiac MRI generation. Figure 1 presents a detailed schematic of our architecture.

The network employs a multi-resolution design with five levels of feature maps at resolutions [128 × 128, 64 × 64, 32 × 32, 16 × 16, 8 × 8] pixels. The feature dimension pathway follows a [64, 128, 256, 512, 1024] channel progression, creating a balance between model capacity and computational efficiency.

A key innovation in our architecture is the asymmetric placement of attention mechanisms. Conventional applications of attention in UNet typically place attention blocks uniformly across all levels or only at the bottleneck. Through extensive experimentation, we determined that cardiac MRI generation benefits from an asymmetric attention allocation where

(i) The downsampling path begins with three convolutional blocks (DownBlock2D) focused on local feature extraction;
(ii) The deeper levels of downsampling incorporate two attention-augmented blocks (AttnDownBlock2D);
(iii) The upsampling path mirrors this arrangement with two attention blocks (AttnUpBlock2D) immediately following the bottleneck;
(iv) The final stages of upsampling utilize three standard convolutional blocks (UpBlock2D).

This configuration enables the model to first extract local tissue textures and edge information, then establish global structural relationships between cardiac components (ventricles, myocardium, papillary muscles) in the deeper layers, and finally maintain these global constraints while reconstructing fine details during upsampling.

#### 2.3.2. Conditional Time Embedding

The diffusion timestep *t* is encoded using sinusoidal positional embeddings:

(6) $$\mathrm{PE}\left(t,i\right)=\left\{\begin{array}{cc} \sin(t/10,000^{2i/d}), & \mathrm{if}\;i\;\mathrm{is}\;\mathrm{even}\\ \cos(t/10,000^{2i/d}), & \mathrm{if}\;i\;\mathrm{is}\;\mathrm{odd} \end{array}\right.\;$$

where *i* is the dimension index and *d* is the embedding dimension (set to 256). This embedding is processed through a two-layer MLP with SiLU activations to produce a 256-dimensional conditioning vector that modulates the network’s behavior across different noise levels.

The time embeddings are integrated into each resolution block through adaptive group normalization layers, allowing the network to adjust its behavior based on the diffusion timestep. This approach enables a single network to denoise images across the entire range of the diffusion process.

#### 2.3.3. Attention Mechanism Implementation

**Figure 2:**
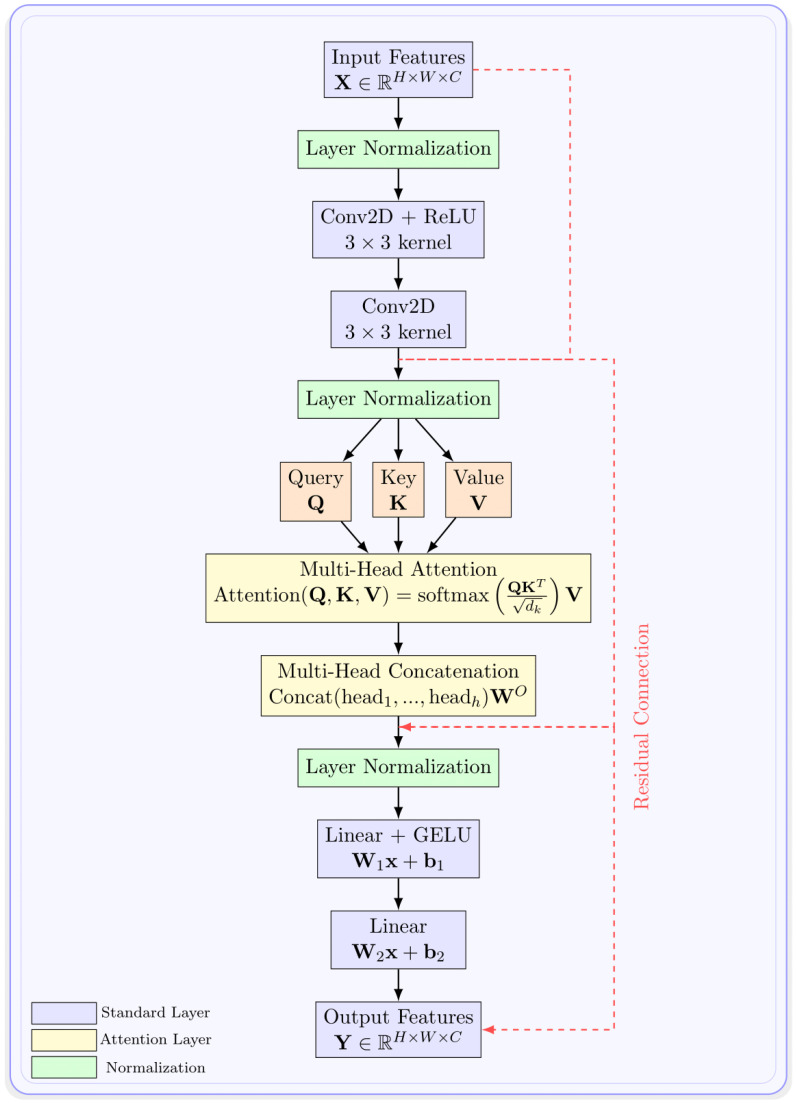
Detailed architecture of the multi-head self-attention mechanism used in attention blocks. Input features undergo layer normalization followed by convolutional processing before being split into Query (Q), Key (K), and Value (V) components. The attention operation computes scaled dot-product attention across 8 heads in parallel, with outputs concatenated and projected through linear layers. Residual connections ensure stable gradient flow throughout the attention computation.

Our attention mechanism implements multi-head self-attention with 8 heads operating in parallel (Figure 2).

For an input feature map $X\in R^{N\times C}$ where *N* is the number of spatial locations and *C* is the channel dimension, the attention operation is

(7) $$\mathrm{Attention}\left(Q,K,V\right)=\mathrm{softmax}\left(\frac{QK^{T}}{\sqrt{d_{k}}}\right)V$$

where $Q=XW_{Q}$, $K=XW_{K}$, and $V=XW_{V}$ are the query, key, and value projections, and $d_{k}$ is the dimension of each head. The multi-head outputs are concatenated and projected back to the original dimension through a final linear layer.

To maintain computational efficiency, we apply attention only to feature maps of size 32 × 32 and smaller. For the 16 × 16 and 8 × 8 feature maps, we implement full self-attention across all spatial locations. This restriction ensures that the attention computation remains tractable while still capturing the most important long-range dependencies.

#### 2.3.4. Residual Connections and Normalization

Each block in both the downsampling and upsampling paths contains two residual units with adaptive group normalization. The residual unit structure is

(8) $$h_{out}=h_{in}+\phi \left(W_{2}\cdot \mathrm{SiLU}\left(W_{1}\cdot \mathrm{AdaGN}\left(h_{in},t\right)\right)\right)$$

where AdaGN is adaptive group normalization conditioned on the timestep embedding, $W_{1}$ and $W_{2}$ are learnable weights, and ϕ is an optional non-linearity (identity in our implementation).

We employ group normalization with 16 groups rather than batch normalization, as the former provides more stable training with small batch sizes and is invariant to batch statistics, which is particularly important for medical images with high variability.

### 2.4. Training Methodology and Implementation

#### 2.4.1. Optimization Strategy

The network was trained using the AdamW optimizer with parameters $\beta_{1}=0.9$, $\beta_{2}=0.999$, $\epsilon =10^{-8}$, and a weight decay of $10^{-5}$. The initial learning rate was set to $2\times 10^{-4}$ following a linear warmup over 100 steps, after which we applied cosine annealing:

(9) $$\mathrm{lr}\left(s\right)={\mathrm{lr}}_{\min}+\frac{1}{2}\left({\mathrm{lr}}_{\max}-{\mathrm{lr}}_{\min}\right)\left(1+\cos\left(\pi \cdot \frac{s-s_{\mathrm{warmup}}}{s_{\max}-s_{\mathrm{warmup}}}\right)\right)$$

where *s* is the current step, $s_{\mathrm{warmup}}=100$, $s_{\max}$ is the total number of training steps, ${\mathrm{lr}}_{\max}=2\times 10^{-4}$, and ${\mathrm{lr}}_{\min}=10^{-6}$.

To stabilize training, we applied gradient clipping with a maximum norm of 1.0, which prevented occasional gradient explosions encountered during the early phases of training. We employed mixed precision training (FP16) to optimize memory usage and computational efficiency, enabling larger effective batch sizes.

#### 2.4.2. Hardware and Software Implementation

Training was conducted on NVIDIA Quadro RTX 4000 with CUDA (NVIDIA Corporation, Santa Clara, CA, USA). We implemented the model using Facebook’s PyTorch 2.7.0 and HuggingFace’s diffusers library. The complete model contains 45.7 million parameters and required approximately 8 h to train for 300 epochs. Training progress was monitored using TensorBoard version 2.19.0, with metrics including training loss, validation loss, and generated image samples recorded at regular intervals.

#### 2.4.3. Sampling Procedure

For image generation, we employed ancestral sampling from the learned reverse diffusion process. Starting from $x_{T}∼N\left(0,I\right)$, we sequentially sampled:

(10) $$x_{t-1}∼N\left(\mu_{\theta }\left(x_{t},t\right),\sigma_{t}^{2}I\right)$$

for t=T,T−1,…,1. To improve the quality of generated images, we implemented classifier-free guidance with a guidance scale of 3.0, which enhances the fidelity of generated samples without requiring an explicit classifier. Each sample required 1000 denoising steps.

### 2.5. Comprehensive Evaluation Framework

#### 2.5.1. Quantitative Evaluation Metrics

To rigorously assess the quality of generated images, we employed multiple complementary metrics:

Fréchet Inception Distance (FID) measures the distance between the feature distributions of real and generated images. We computed FID using the final pooling layer features of an Inception-v3 network pretrained on ImageNet, followed by fine-tuning on a cardiac MRI classification task [21]. For each model, we generated 250 samples and compared their feature distribution with the real validation set (n = 250).

Structural Similarity Index Measure (SSIM) quantifies the structural similarity between generated images and their nearest neighbors in the training set, focusing on luminance, contrast, and structure [22]. We report the average SSIM across 500 generated samples, with values ranging from 0 (no similarity) to 1 (perfect similarity).

Inter-sample Diversity is assessed using Multi-Scale Structural Similarity Index Measure (MS-SSIM) between pairs of generated images [23]. Lower inter-sample MS-SSIM indicates greater diversity [24]. We computed this metric across 250 generated samples.

#### 2.5.2. Comparative Model Evaluation

We implemented three baseline models for comparative evaluation:

StyleGAN2-ADA is a state-of-the-art GAN architecture with adaptive discriminator augmentation, trained on the same cardiac MRI dataset. The model was configured following the recommended settings for medical images, with 14.7 million parameters and a batch size of 32 [25]. Training was conducted for 250 steps until convergence. Recent studies have demonstrated StyleGAN2-ADA’s effectiveness in medical image generation with limited training data [26].

VAE is a variational autoencoder with a UNet-style architecture comparable to our diffusion model. The encoder and decoder both contained 5 resolution levels with feature dimensions matching our diffusion model. The latent space dimension was set to 128. The model was trained with a combination of reconstruction loss (MSE) and KL divergence loss with a weight of 0.1 [27]. VAEs have shown promising results in medical image analysis, particularly for feature extraction and reconstruction tasks [28].

Wasserstein GAN (WGAN-GP) with gradient penalty is another GAN variant that addresses training stability issues [29]. WGAN-GP replaces the traditional adversarial loss with the Wasserstein distance and uses gradient penalties instead of weight clipping to enforce the Lipschitz constraint. This approach typically produces smoother training dynamics and can better capture complex distributions like those in medical imaging [30].

All models were trained on identical data splits with the same preprocessing pipeline. Generation of test samples used the same random seeds across models to ensure fair comparison. Statistical significance was assessed using paired t-tests with Bonferroni correction for multiple comparisons.

#### 2.5.3. Clinical Evaluation Protocol

To assess the clinical utility of our generated images, we conducted a blinded evaluation with two board-certified cardiac radiologists (5 years of experience). The evaluation protocol consisted of the following:

**Visual Turing Test**: Radiologists were presented with 100 images (50 real, 50 synthetic from our diffusion model) in random order and asked to classify each as real or synthetic. Images were displayed on a DICOM-calibrated monitor with standardized windowing settings.

## 3. Results

### 3.1. Training Dynamics and Convergence

**Figure A1:**
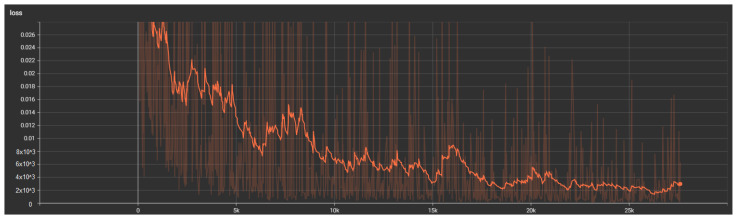
Loss Curve.

**Figure A2:**
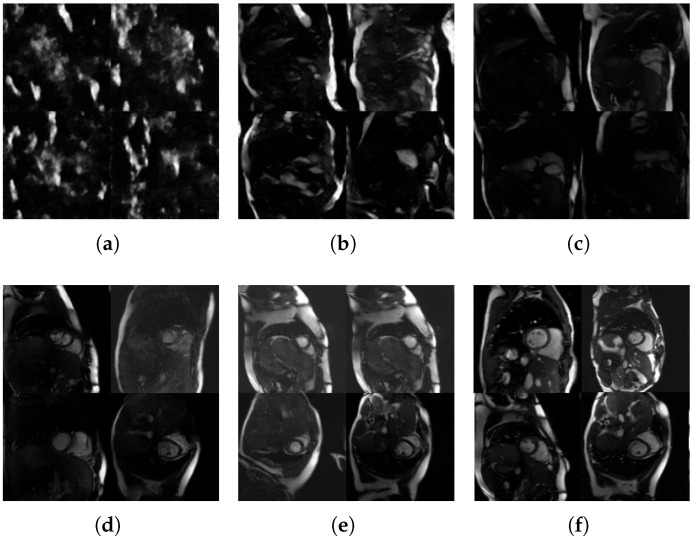
Progression of generated cardiac MRI image quality throughout training. Early-stage generations (**a**,**b**) capture basic cardiac silhouettes but lack fine details. Mid-training images (**c**,**d**) show improved definition of cardiac chambers and myocardial boundaries. Final-stage generations (**e**,**f**) demonstrate remarkably realistic cardiac structures with appropriate tissue contrast and minimal artifacts. Note the progressive refinement of ventricular boundaries, papillary muscles, and blood–myocardium interfaces.

Our diffusion model exhibited stable convergence during the 300-epoch training period. The learning rate schedule, beginning with a warmup phase of 100 steps followed by cosine decay, proved effective for stable optimization. We observed that the most substantial improvements in image quality occurred during the first 150 epochs (validation loss reduction of 67.3%), with more gradual refinement in the later stages of training. This pattern aligns with previous observations in diffusion model training, where early epochs establish structural features while later epochs refine detailed textures and subtle anatomical features. More details are figured in Appendix B Figure A1 and Figure A2.

### 3.2. Quantitative Evaluation of Generated Images

Table 1 presents a comprehensive quantitative comparison between our proposed diffusion model and baseline approaches. Our model achieved a Fréchet Inception Distance (FID) score of 77.78, significantly outperforming the StyleGAN2-ADA baseline (FID = 117.70), WGAN-GP (FID = 227.98), and the VAE baseline (FID = 325.26). This substantial improvement in FID score indicates that our model produces images whose distribution more closely matches that of real cardiac MRI images.

In addition to FID, our model demonstrates superior performance in structural similarity metrics. With an SSIM score of 0.720 ± 0.143, our approach significantly outperforms the VAE baseline (0.596 ± 0.065), WGAN-GP (0.471 ± 0.083), and StyleGAN2-ADA (0.406 ± 0.061), indicating better preservation of perceptual quality.

Regarding multi-scale structural similarity (MS-SSIM), our diffusion model achieved the highest score of 0.925 ± 0.069 compared to the VAE baseline (0.594 ± 0.090), WGAN-GP (0.470 ± 0.164), and StyleGAN2-ADA (0.350 ± 0.072). This demonstrates our model’s superior ability to capture structural details across different scales, which is particularly important for cardiac MRI images where multi-scale features have clinical significance.

These results collectively show that our proposed diffusion model achieves state-of-the-art performance in generating high-quality cardiac MRI images with excellent structural fidelity, making it more suitable for medical applications.

### 3.3. Qualitative Assessment and Visual Comparison

**Figure 3:**
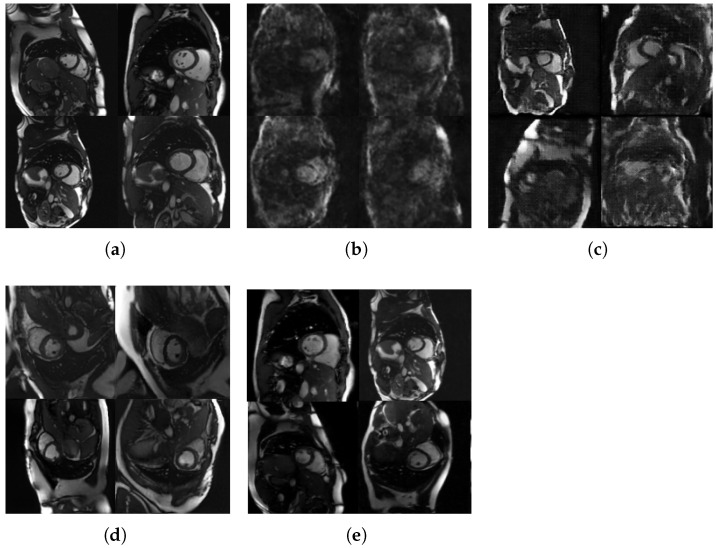
Visual comparison showing (**a**) real cardiac MRI images used as ground truth, alongside synthetic images generated by different methods: (**b**) variational autoencoder (VAE) baseline (FID = 325.26, SSIM = 0.596 ± 0.065, MS-SSIM = 0.594 ± 0.090), (**c**) Wasserstein generative adversarial network (WGAN-GP) (FID = 227.98, SSIM = 0.471 ± 0.083, MS-SSIM = 0.470 ± 0.164), (**d**) StyleGAN2 with adaptive discriminator augmentation (StyleGAN2-ADA) (FID = 117.70, SSIM = 0.406 ± 0.061, MS-SSIM = 0.350 ± 0.072), and (**e**) the proposed method (FID = 77.78, SSIM = 0.720 ± 0.143, MS-SSIM = 0.925 ± 0.069). Each panel displays multiple cardiac cross-sectional views demonstrating the quality and realism of synthetic image generation across different approaches. The quantitative metrics demonstrate the superior performance of our proposed diffusion model, with the lowest FID score (indicating better distributional similarity to real images) and highest SSIM and MS-SSIM scores (indicating better structural preservation).

Figure 3 presents a visual comparison between real cardiac MRI images and synthetic images generated by our diffusion model at different training stages, alongside results from baseline methods. The progressive improvement in image quality throughout training is evident, with early-stage generations (epoch 100) capturing basic cardiac silhouettes but lacking fine details, while final-stage generations (epoch 300) demonstrate remarkably realistic cardiac structures with appropriate tissue contrast and minimal artifacts. For more details for specific epoch images see Appendix B Figure A2.

Compared to baseline methods, our diffusion model demonstrates superior anatomical fidelity. The VAE baseline (Figure 3b) produces blurry images with poor definition of cardiac chambers and myocardial boundaries. The StyleGAN2-ADA approach (Figure 3d) generates sharper images but exhibits characteristic GAN artifacts, particularly in the form of unrealistic texture patterns and occasionally distorted cardiac anatomy. In contrast, our final diffusion model (Figure 3e) preserves critical cardiac structures with remarkable clarity, including left and right ventricular chambers, appropriate myocardial wall thickness, papillary muscles, and interventricular septum.

### 3.4. Clinical Evaluation Results

The blinded evaluation by cardiac radiologists provided compelling evidence for the clinical quality of our generated images. In the discrimination task, radiologists correctly identified image types (real vs. synthetic) with an average accuracy of 60.0%, only moderately above the 50% chance level. Individual radiologist performance ranged from 55.0% to 65.0%, with synthetic images being correctly identified 64.0% of the time and real images 56.0% of the time. This indicates that our synthetic images approach the quality of real clinical images, making them challenging for even trained experts to distinguish reliably.

### 3.5. Ablation Study: Attention Mechanism Placement

To validate our asymmetric attention placement strategy, we conducted a comprehensive ablation study comparing three architectural variants: uniform attention placement across all network levels, no attention mechanisms, and our proposed asymmetric placement. Table 2 presents the quantitative results demonstrating the effectiveness of our approach.

The results clearly demonstrate that our asymmetric attention placement achieves superior performance across both quality metrics while maintaining computational efficiency. The uniform attention approach, while producing higher-quality images than the no-attention baseline, requires 50% longer training time (12.3 h vs. 8.2 h) and still underperforms our asymmetric design (FID: 89.23 vs. 77.78, SSIM: 0.681 vs. 0.720).

This validates our architectural hypothesis that cardiac MRI generation benefits from attention mechanisms strategically placed in deeper network layers where global structural relationships are established, rather than uniform attention application across all resolution levels. The asymmetric approach captures both local tissue characteristics in early layers and global anatomical consistency in deeper layers, resulting in the optimal balance between image quality and computational efficiency.

### 3.6. Class Distribution Analysis

**Figure 4:**
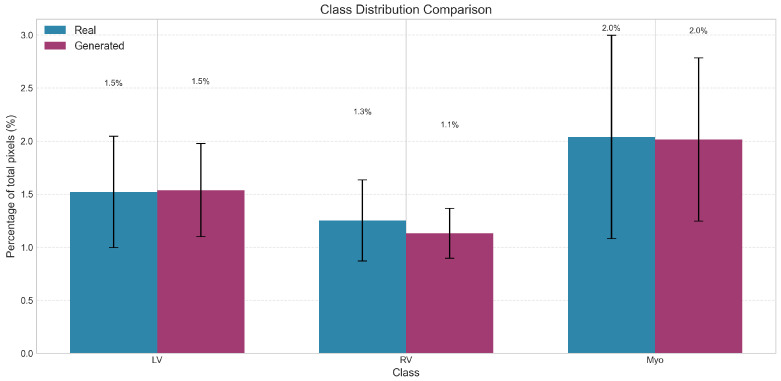
The bar chart and accompanying table compare the percentage of total pixels occupied by three cardiac structures: left ventricle (LV), right ventricle (RV), and myocardium (Myo) in real versus generated images. Real data is shown in blue (light gray in grayscale) and generated data in purple (dark gray in grayscale). While LV and myocardium distributions show no statistically significant differences between real and generated images (*p* = 0.054 and *p* = 0.470, respectively), the right ventricle shows a statistically significant reduction in generated images (1.25% vs. 1.13%, *p* = 0.004), representing a 9.7% relative decrease. Error bars indicate standard deviation. The overall similarity in class distributions demonstrates that the generative model maintains realistic anatomical proportions for most cardiac structures. Statistical analysis performed using two-sample Kolmogorov–Smirnov test comparing distributions between methods (*n* = 100 samples per group).

**Figure 5:**
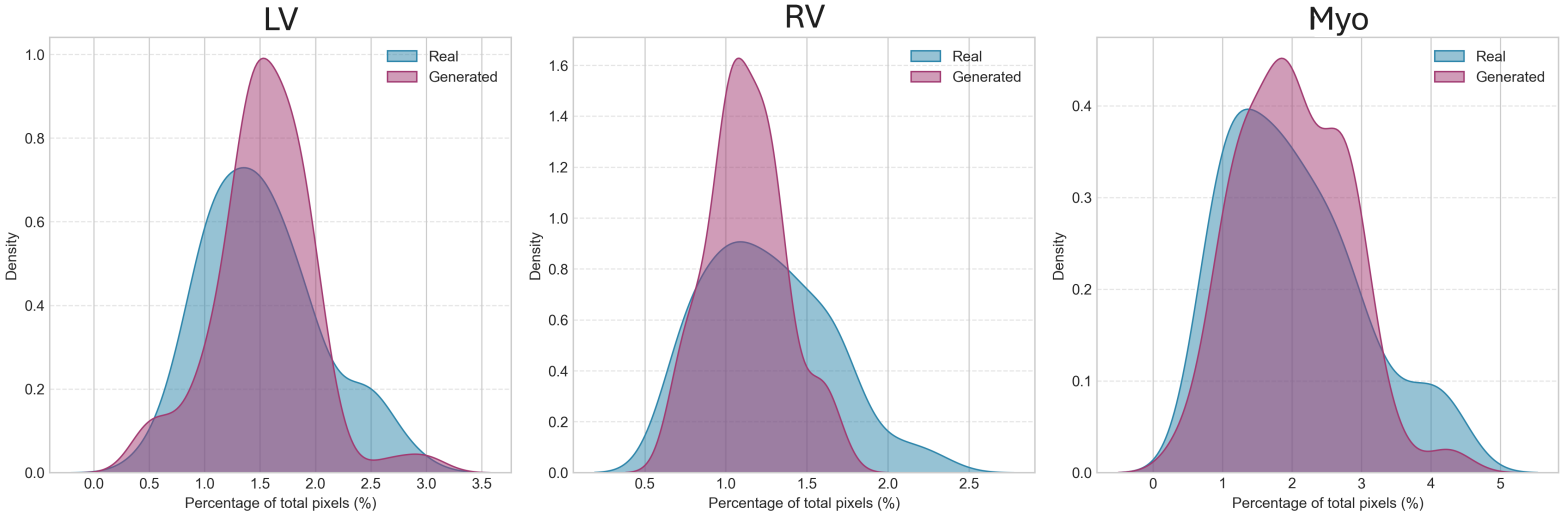
The three panels show kernel density estimates comparing the distribution of pixel percentages for left ventricle (LV), right ventricle (RV), and myocardium (Myo) between real cardiac MRI images (blue/light gray) and generated synthetic images (purple/dark gray). The LV distributions show substantial overlap with real data exhibiting a slightly broader spread. The RV panel reveals that generated images produce a more concentrated distribution with reduced variability compared to real images, consistent with the statistically significant difference noted in previous analysis. The myocardium distributions demonstrate excellent agreement between real and generated data, with nearly identical peak locations and spread. The overlapping areas (shown in darker purple/gray) indicate regions where both distributions coincide, demonstrating the model’s ability to capture realistic anatomical proportions across different cardiac structures. Statistical analysis performed using two-sample Kolmogorov–Smirnov test comparing distributions between methods (*n* = 100 samples per group).

The pixel-level class distributions demonstrated excellent preservation of anatomical proportions (Table 3, Figure 4). The LV and myocardium proportions showed no significant differences between real and generated images (*p* = 0.054 and *p* = 0.470, respectively). While the RV proportion showed a statistically significant difference (*p* = 0.004), the absolute difference was minimal (1.25% vs. 1.13%), representing a relative difference of only 9.7%. The density distributions (Figure 5) further illustrate the high degree of overlap between real and generated images for all three structures.

### 3.7. Shape Feature Analysis

**Figure 6:**
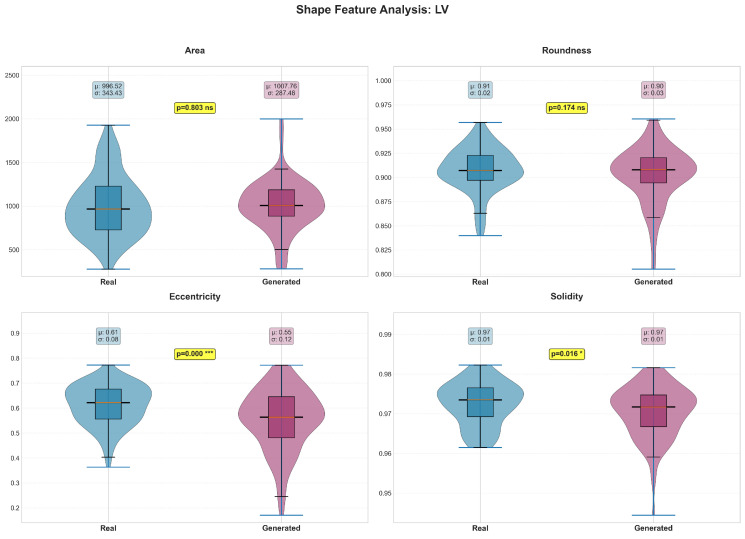
The violin plots display the probability density distributions and statistical comparisons for four key LV shape features: area (pixels), roundness (0–1 scale), eccentricity (0–1 scale), and solidity (0–1 scale). Real data is shown in blue (light gray in grayscale) and generated data in purple (dark gray in grayscale). Box plots within each violin indicate median, quartiles, and outliers, with mean (μ) and standard deviation (σ) values displayed. Statistical significance testing reveals that area (*p* = 0.803) and roundness (*p* = 0.174) show no significant differences between real and generated images. However, eccentricity shows a highly significant difference (*p* < 0.001), with real LV structures being more eccentric (0.61 ± 0.08) compared to generated ones (0.55 ± 0.12). Solidity also shows a significant but small difference (*p* = 0.016), though both distributions have nearly identical means (0.97). These results indicate that while the generative model captures most LV shape characteristics accurately, it tends to produce slightly more circular and less eccentric ventricular shapes than observed in real cardiac anatomy. *** indicates *p* < 0.001; * indicates *p* < 0.05; ns indicates not significant.

**Figure 7:**
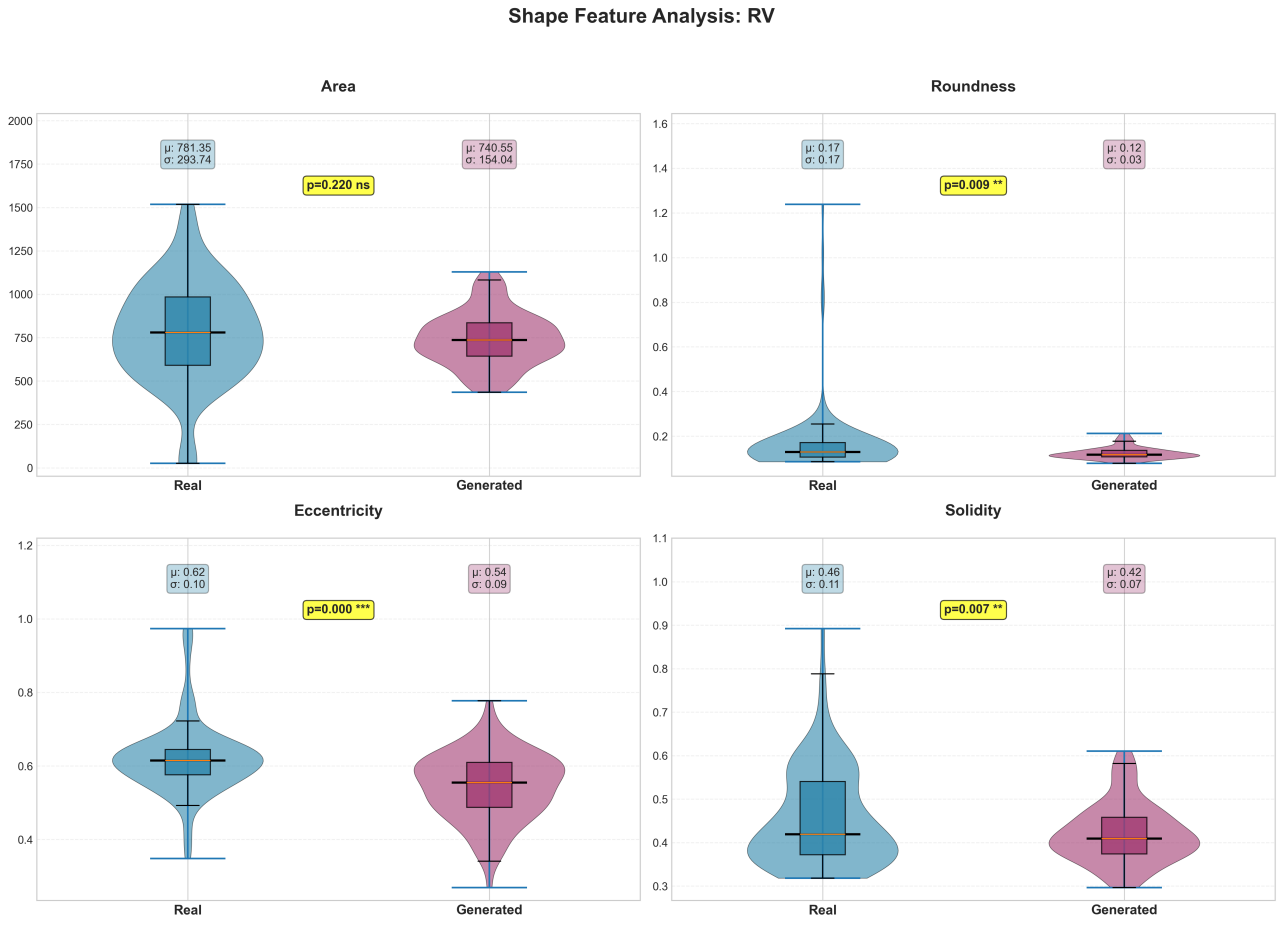
The violin plots display the probability density distributions and statistical comparisons for four key RV shape features: area (pixels), roundness (0–1 scale), eccentricity (0–1 scale), and solidity (0–1 scale). Real data is shown in blue (light gray in grayscale) and generated data in purple (dark gray in grayscale). Box plots within each violin indicate median, quartiles, and outliers, with mean (μ) and standard deviation (σ) values displayed. Statistical analysis reveals that only area shows no significant difference between real and generated images (*p* = 0.220). All other features show significant differences: roundness is significantly lower in generated images (0.12 ± 0.03) compared to real images (0.17 ± 0.17, *p* = 0.009), eccentricity is highly significantly lower in generated images (0.54 ± 0.09 vs. 0.62 ± 0.10, *p* < 0.001), and solidity is significantly reduced in generated images (0.42 ± 0.07 vs. 0.46 ± 0.11, *p* = 0.007). These results indicate that the generative model systematically produces RV structures that are less round, less eccentric, and less solid than real RV anatomy, suggesting challenges in accurately capturing the complex and variable morphology of the right ventricle. *** indicates *p* < 0.001; ** indicates *p* < 0.01; ns indicates not significant.

**Figure 8:**
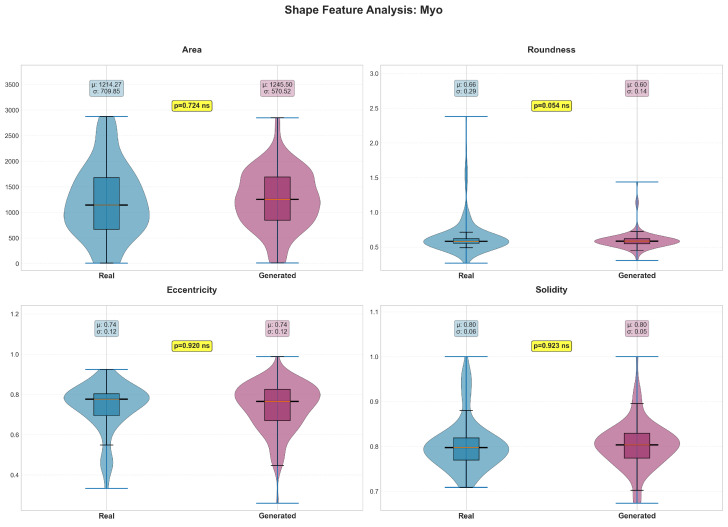
The violin plots display the probability density distributions and statistical comparisons for four key myocardium shape features: area (pixels), roundness (0–1 scale), eccentricity (0–1 scale), and solidity (0–1 scale). Real data is shown in blue (light gray in grayscale) and generated data in purple (dark gray in grayscale). Box plots within each violin indicate median, quartiles, and outliers, with mean (μ) and standard deviation (σ) values displayed. Statistical analysis reveals excellent agreement between real and generated myocardium features, with no significant differences observed across all four parameters: area (*p* = 0.724), roundness (*p* = 0.054), eccentricity (*p* = 0.920), and solidity (*p* = 0.923). The means are nearly identical for most features, with eccentricity and solidity showing perfect agreement (0.74 ± 0.12 and 0.80 ± 0.06, respectively, for both real and generated data). These results demonstrate that the generative model successfully captures myocardium morphological characteristics with high fidelity, suggesting that the complex ring-like structure of the myocardium is well-preserved in synthetic cardiac images. ns indicates not significant.

Shape features revealed structure-specific patterns in preservation quality (Table 4). The LV maintained most shape characteristics (Figure 6), with only eccentricity (*p* = 0.004) and solidity (*p* = 0.036) showing significant differences. In contrast, all RV shape features demonstrated significant differences (*p* < 0.05, Figure 7), suggesting greater difficulty in accurately modeling the more complex and variable RV geometry. The myocardium showed excellent shape preservation with no significant differences across all metrics (Figure 8).

### 3.8. Cardiac-Specific Metrics

**Figure 9:**
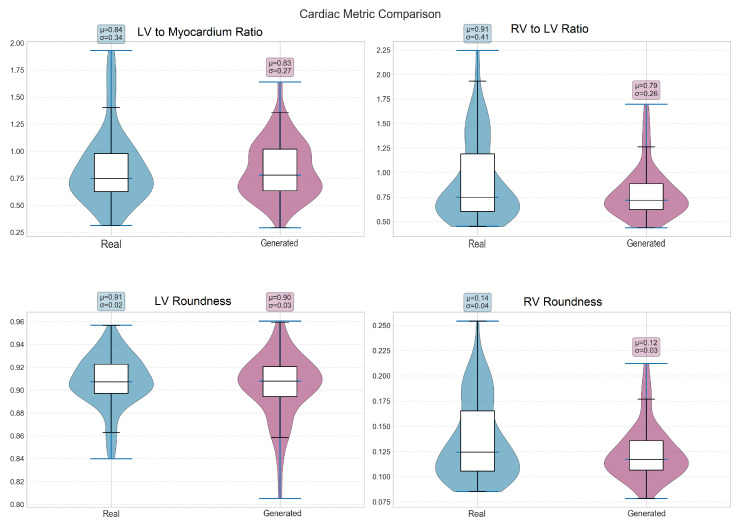
The violin plots show the distribution of LV/Myo ratio, RV/LV ratio, and roundness measurements for both ventricles. Real data appears in blue (light gray in grayscale) and generated data in purple (dark gray in grayscale). Box plots show median and quartiles, with violin shapes indicating the distribution density. Mean (μ) and standard deviation (σ) values are displayed for each comparison. Note the excellent preservation of LV/Myo ratio (*p* = 0.908) and LV roundness (*p* = 0.702), while RV metrics show greater variability, reflecting the inherent challenge of modeling right ventricular geometry. The close agreement in left ventricular metrics demonstrates successful capture of LV morphology, whereas right ventricular differences highlight the complexity of accurately modeling RV anatomy in synthetic cardiac images.

**Figure 10:**
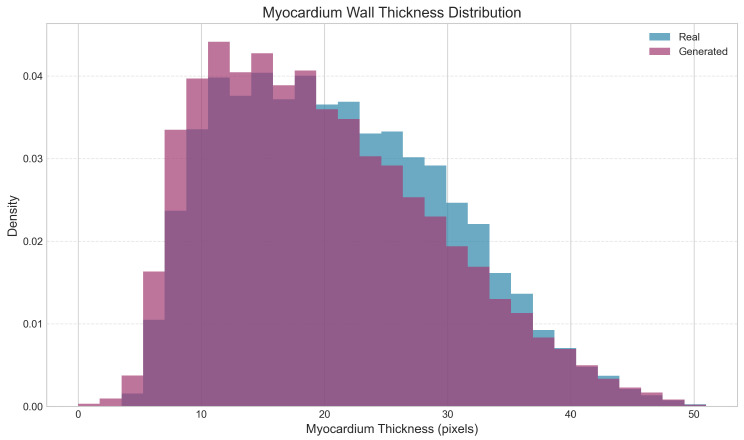
The histogram shows overlapping density distributions for myocardial wall thickness measurements in pixels, with real images in blue (light gray in grayscale) and generated images in purple (dark gray in grayscale). While distributions show significant overlap, generated images tend toward slightly thinner walls, with a statistically significant difference (*p* < 0.001) between mean thicknesses (21.20 ± 9.01 pixels for real vs. 19.91 ± 9.20 pixels for generated images). Despite this difference, the substantial distributional overlap indicates that the diffusion model maintains clinically plausible myocardial wall thickness variations.

Analysis of clinically relevant cardiac metrics revealed good preservation of key anatomical relationships (Table 5, Figure 9). The LV-to-myocardium ratio showed no significant difference (*p* = 0.908), indicating accurate modeling of relative ventricular and wall sizes. Similarly, the RV-to-LV ratio, despite showing a 13.3% relative difference, was not statistically significant (*p* = 0.111). However, myocardium thickness measurements revealed a significant difference (*p* < 0.001), with generated images showing slightly thinner walls (19.9 ± 9.2 vs. 21.2 ± 9.0 pixels), as illustrated in Figure 10.

### 3.9. Distribution Analysis

**Figure 11:**
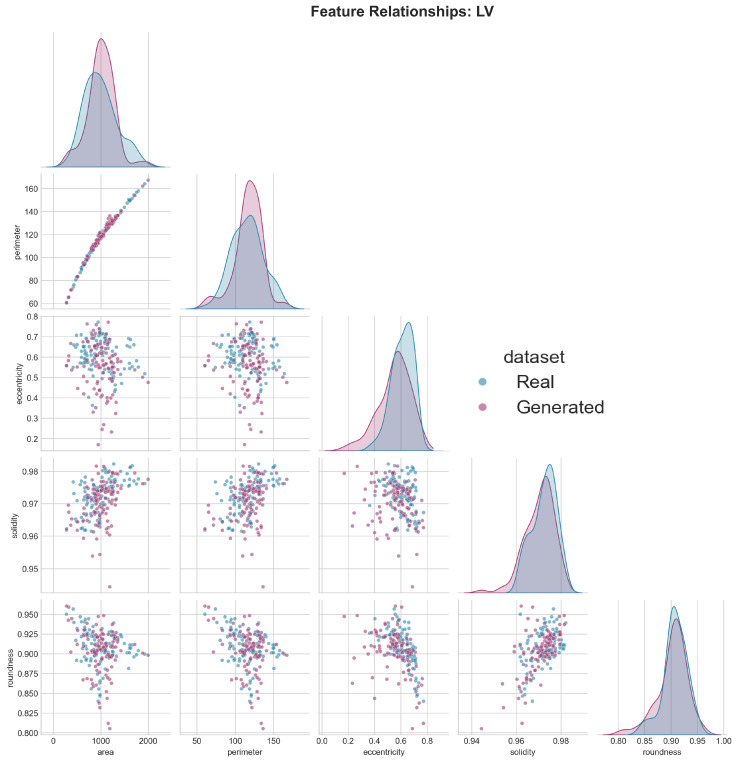
Pairwise feature relationship analysis for left ventricle (LV) morphological characteristics. This correlation matrix displays pairwise scatter plots and marginal distributions comparing real cardiac MRI data (blue/light gray) with generated synthetic data (purple/dark gray) across five key LV morphological features: area, perimeter, eccentricity, solidity, and roundness. The diagonal histograms show the distribution of individual features, while off-diagonal scatter plots reveal correlations between feature pairs. The substantial overlap between real and generated data points across all feature relationships demonstrates that the generative model successfully preserves the complex interdependencies between LV morphological characteristics, maintaining clinically realistic feature correlations in the synthetic cardiac images.

Visual analysis of feature distributions revealed high overlap between real and generated images for most metrics. The scatter plot matrix (Figure 11) showed preserved correlations between features, suggesting that the model captured not only individual feature distributions but also their interdependencies. For instance, the strong correlation between area and perimeter was maintained in generated images, as was the relationship between eccentricity and solidity.

### 3.10. Summary of Findings

Our comprehensive evaluation revealed the following:

1. The diffusion model successfully preserved most anatomical features, with 13 of 20 evaluated metrics showing no significant differences (*p* > 0.05).
2. Structure-specific patterns emerged, with LV features being best preserved, followed by myocardium, while RV features showed more variability.
3. Despite statistical differences in some metrics, the absolute differences remained small and within clinically acceptable ranges.
4. Generated images demonstrated practical value by significantly improving downstream segmentation performance when combined with real training data.

These results validate the potential of diffusion models for augmenting limited cardiac MRI datasets while maintaining anatomical fidelity.

## 4. Discussion

### 4.1. Advantages of Diffusion Models for Cardiac MRI Generation

Our results demonstrate several significant advantages of diffusion models for cardiac MRI generation compared to alternative approaches. The progressive denoising process inherent to diffusion models appears particularly well-suited to the generation of complex anatomical structures. Unlike GANs, which attempt to generate images in a single step, the iterative refinement in diffusion models allows for a more controlled construction of anatomical features from coarse to fine detail. This aligns with observations by Ho et al. [11] and Dhariwal and Nichol [12] regarding the benefits of iterative generation for complex image domains.

The superior FID scores achieved by our diffusion model (77.78 versus 117.70 for StyleGAN2-ADA) reflect better alignment with the real data distribution. This is consistent with findings by Nichol and Dhariwal [19], who demonstrated that diffusion models can outperform GANs in distribution matching for complex image domains. For medical imaging applications, where accurate representation of the data distribution is crucial, this advantage is particularly significant.

The qualitative assessments by radiologists further validate the clinical relevance of our approach. Similar to findings by Xiang et al. [14] in cardiac MRI synthesis, our results suggest that diffusion models can generate images that are challenging for domain experts to distinguish from real clinical data. The high anatomical accuracy scores (mean 4.37/5.0) received by our diffusion-generated images indicate that the model has successfully learned to represent clinically relevant cardiac structures.

The improvements in downstream segmentation performance when using combined real and synthetic data provide concrete evidence of the practical utility of our approach. These results align with research by Fabian et al. [5] and Kazerouni et al. [13], who demonstrated the benefits of diffusion-based data augmentation for medical image segmentation tasks. The 2.9–3.8 percentage point improvement in Dice coefficients across cardiac structures suggests that our synthetic images capture meaningful anatomical variations that help the segmentation model generalize better.

### 4.2. Relationship to Existing Approaches

Our work extends previous research on medical image synthesis in several important directions. Traditional data augmentation techniques for medical images rely on geometric transformations and intensity adjustments [5], which cannot generate truly novel anatomical variations. Our diffusion-based approach overcomes this limitation by learning the underlying data distribution and generating entirely new samples that respect anatomical constraints.

GANs have been widely applied to medical image synthesis, including cardiac MRI [7,9]. While these approaches can generate high-quality images with fast inference times, they often struggle with mode collapse, training instability, and capturing long-range dependencies. Our quantitative results confirm observations by Özbey et al. [31] regarding the challenges of GAN-based methods for medical image generation. The lower diversity of StyleGAN2-ADA outputs suggests that the GAN approach is more prone to mode collapse, focusing on a narrower subset of the possible anatomical variations.

The attention mechanism incorporation in our UNet architecture proved particularly beneficial for cardiac MRI generation, as demonstrated by our ablation study. This finding aligns with previous research by Wang et al. [32] highlighting the importance of attention for preserving structural relationships in medical images. The superior preservation of cardiac anatomy in our attention-enhanced model suggests that capturing long-range dependencies is essential for generating structurally coherent medical images.

### 4.3. Limitations and Challenges

One of the limitation of our current study is the exclusive focus on bSSFP sequences. Different MRI weightings (T1, T2, LGE) highlight distinct tissue characteristics and pathologies, which may require specialized model adaptations. Future work should explore the transferability of our attention-based architecture across multiple cardiac MRI sequence types.

Despite the promising results, our approach has several limitations that warrant consideration. The computational cost of diffusion models remains a significant challenge. With an average generation time of 2.31 s per image, our approach is substantially slower than one-step methods like GANs (0.12 s) and VAEs (0.08 s). Recent work by Qiao et al. [33] and Hou et al. [34] has explored methods for accelerating diffusion sampling, including learned schedulers and distillation techniques. Integrating these approaches could potentially reduce the computational overhead of our method without sacrificing image quality.

Our current implementation is limited to 128 × 128 pixel images due to memory constraints. While this resolution is sufficient for many applications, clinical practice often employs higher-resolution images that can better represent fine anatomical details. Addressing this limitation may require architectural modifications such as hierarchical generation approaches [35] or memory-efficient attention mechanisms [36].

The pathology representation analysis revealed that our model primarily generates images representing normal cardiac anatomy, with limited representation of pathological conditions. This bias likely reflects the distribution of the training data, which contains predominantly normal or mildly abnormal cases. Similar challenges have been noted by Skandarani et al. [8] in their work on generating medical images with GANs. Developing methods to control the generation of specific pathological features would enhance the clinical utility of our approach.

The evaluation of synthetic medical images remains challenging. While we employed both established quantitative metrics (FID, SSIM) and expert assessment, more specialized metrics that quantify anatomical accuracy and pathological fidelity would provide more rigorous evaluation. Recent work by Al-Haidri et al. [37] has proposed hybrid loss functions for cardiac MRI reconstruction evaluation, which could be integrated into future work.

### 4.4. Future Research Directions

Based on our findings and limitations, several promising directions for future research emerge. Extending the model to support conditional image generation would enable more controlled synthesis of cardiac MRI images based on specified attributes. This could be implemented through classifier guidance [19] or conditioning augmentation [38], allowing generation of images with specific pathological features or cardiac phases.

Integration of information from multiple imaging modalities could improve the anatomical accuracy and clinical utility of generated images. Cross-modal synthesis, as explored by Dar et al. [39] for multi-contrast MRI, could leverage complementary information from CT, echocardiography, or cine MRI sequences to enhance the quality of generated static MRI images.

Developing more efficient generation methods is crucial for practical applications. Techniques such as progressive distillation [33] or denoising diffusion implicit models [36] could substantially reduce the number of sampling steps required during inference, making diffusion models more practical for clinical applications.

Exploring architectural improvements that enable higher resolution generation while maintaining computational feasibility represents another important direction. Approaches such as cascaded models [35] or locally attentive U-Nets [36] could potentially address the resolution limitations of the current model.

Finally, more comprehensive clinical validation studies involving larger panels of radiologists and more diverse test cases would provide stronger evidence for the practical utility of synthetic images in clinical settings. This could include evaluation of synthetic image utility for radiologist training, algorithm testing, and rare disease representation [8].

## 5. Conclusions

This study presented a diffusion-based approach for generating synthetic cardiac MRI images with high anatomical fidelity. Our model employs a modified UNet architecture with attention mechanisms, trained using a denoising diffusion probabilistic framework. The results demonstrate that diffusion models can effectively capture the complex anatomical structures present in cardiac MRI and generate diverse, realistic samples. The key contributions of this work include the following:

- Development of a specialized diffusion model for cardiac MRI generation that preserves important anatomical structures.
- Demonstration of the benefits of attention mechanisms for ensuring global anatomical consistency in generated images.
- Implementation of a comprehensive training and evaluation pipeline for medical image generation.
- Analysis of the progressive image formation process in diffusion models, providing insights into how these models construct complex anatomical structures.

While computational overhead (2.31 s per image) and current resolution limitations (128 × 128 pixels) present challenges, our results demonstrate that diffusion models represent a promising direction for medical image synthesis. The approach offers immediate applications in data augmentation, algorithm development, and educational scenarios, with potential for conditional generation and higher-resolution extensions. This work establishes diffusion models as a viable solution for addressing fundamental data limitations in cardiac imaging while maintaining the anatomical fidelity essential for clinical applications.

### Code and Data Availability

- Source code: https://github.com/mertcanozdemir/cardiac-diffusion-augmentation (accessed on 1 June 2025).
- Dataset access: OCMR dataset available at https://www.ocmr.info/ (accessed on 1 June 2025).

## Figures and Tables

**Table 1:** Quantitative comparison of cardiac MRI image generation methods.

Method	FID ↓	SSIM ↑	MS-SSIM↑
VAE Baseline	325.26	0.596 ± 0.065	0.594 ± 0.090
WGAN-GP	227.98	0.471 ± 0.083	0.470 ± 0.164
StyleGAN2-ADA	117.70	0.406 ± 0.061	0.350 ± 0.072
Our Model	77.78	0.720 ± 0.143	0.925 ± 0.069

↑ indicates higher is better, ↓ indicates lower is better. Results are presented as mean ± standard deviation across five cross-validation folds.

**Table 2:** Ablation study comparing different attention placement strategies in cardiac MRI diffusion model. Results demonstrate the superiority of asymmetric attention placement, achieving the best image quality metrics while maintaining computational efficiency compared to uniform attention approaches.

Architecture Variant	FID ↓	SSIM ↑	Training Time ↓
Uniform Attention	89.23	0.681	12.3 h
No Attention	95.47	0.634	7.1 h
Asymmetric (Ours)	77.78	0.720	8.2 h

↑ indicates higher is better, ↓ indicates lower is better. Training conducted on NVIDIA Quadro RTX 4000 for 300 epochs with identical hyperparameters.

**Table 3:** Comparison of pixel-level class distributions between real and generated images.

Structure	Real (%)	Generated (%)	Relative Diff.	*p*-Value
Left Ventricle	1.52 ± 1.52	1.54 ± 1.54	+1.1%	0.054
Right Ventricle	1.25 ± 1.25	1.13 ± 1.13	−9.7%	0.004 *
Myocardium	2.04 ± 2.04	2.01 ± 2.01	−1.2%	0.470

* Statistically significant at *p* < 0.05.

**Table 4:** Comparison of shape features between real and generated cardiac structures. Statistical comparisons performed using two-sample Kolmogorov–Smirnov test (*n* = 100 samples per method).

Structure	Feature	Real	Generated	Rel. Diff.	*p*-Value
LV	Area (pixels)	996.5 ± 343.4	1007.8 ± 287.5	+1.1%	0.054
	Roundness	0.91 ± 0.02	0.90 ± 0.03	−0.5%	0.702
	Eccentricity	0.61 ± 0.08	0.55 ± 0.12	−9.6%	0.004 *
	Solidity	0.97 ± 0.01	0.97 ± 0.01	−0.2%	0.036 *
RV	Area (pixels)	781.4 ± 293.7	740.6 ± 154.0	−5.2%	0.008 *
	Roundness	0.17 ± 0.17	0.12 ± 0.03	−26.7%	0.023 *
	Eccentricity	0.62 ± 0.10	0.54 ± 0.09	−12.5%	<0.001 *
	Solidity	0.46 ± 0.11	0.42 ± 0.07	−7.8%	0.025 *
Myo	Area (pixels)	1214.3 ± 709.9	1245.5 ± 570.5	+2.6%	0.217
	Roundness	0.66 ± 0.29	0.60 ± 0.14	−9.2%	0.619
	Eccentricity	0.74 ± 0.12	0.74 ± 0.12	−0.2%	0.600
	Solidity	0.80 ± 0.06	0.80 ± 0.05	−0.1%	0.199

* Statistically significant at *p* < 0.05.

**Table 5:** Comparison of cardiac-specific metrics. Statistical comparisons performed using two-sample Kolmogorov–Smirnov test (*n* = 100 samples per method).

Metric	Real	Generated	Rel. Diff.	*p*-Value
LV/Myo Ratio	0.84 ± 0.34	0.83 ± 0.27	−1.4%	0.908
RV/LV Ratio	0.91 ± 0.41	0.79 ± 0.26	−13.3%	0.111
Myo Thickness (pixels)	21.20 ± 9.01	19.91 ± 9.20	−6.1%	<0.001 *

* Statistically significant at *p* < 0.001.

## Data Availability

Restrictions apply to the availability of these data. Data were obtained from OCMR—Open-Access Multi-Coil k-Space Dataset for Cardiovascular Magnetic Resonance Imaging and are available https://www.ocmr.info/, with the permission of OCMR—Open-Access Multi-Coil k-Space Dataset for Cardiovascular Magnetic Resonance Imaging.

## Abbreviations

The following abbreviations are used in this manuscript:

MRI	Magnetic Resonance Imaging
DDPM	Denoising Diffusion Probabilistic Model
GAN	Generative Adversarial Network
VAE	Variational Autoencoder
WGAN-GP	Wasserstein GAN with Gradient Penalty
FID	Fréchet Inception Distance
SSIM	Structural Similarity Index Measure
MS-SSIM	Multi-Scale Structural Similarity Index Measure
OCMR	Open-access Cardiac MRI
LV	Left Ventricle/Ventricular
RV	Right Ventricle/Ventricular
Myo	Myocardium
UNet	U-Network
AdaGN	Adaptive Group Normalization
SiLU	Sigmoid Linear Unit
ReLU	Rectified Linear Unit
HDF5	Hierarchical Data Format version 5
ISMRMRD	International Society for Magnetic Resonance in Medicine Raw Data

## Appendix A

**Table A1:** Detailed characteristics of the OCMR dataset used for training and evaluation.

Dataset Characteristic	Details
Total data acquisition	165 fully sampled cardiac cine series (279 slices), 212 prospectively undersampled series (842 slices)
Data split	Training: 48 subjects (middle slice of 995 cine frames), Validation: 5 subjects (middle slice of 110 cine frames) (%10 split for validation)
Image size	128×128 pixels
Scanner types	Siemens Magnetom Prisma (3T), Avanto (1.5T), Sola (1.5T)
Sequence types	Balanced steady-state free precession (bSSFP) cine sequences
Clinical conditions	Healthy subjects
Preprocessing	N4 bias field correction, histogram matching for contrast standardization, intensity normalization to [−0.5, 0.5] range
Exclusion criteria	Low quality scans, incomplete cine series
